# Supplementary material for: All-at-once RNA folding with 3D motif prediction framed by evolutionary information
Source: Nat Methods. 2025 Oct 3;22(10):2094–106. doi: 10.1038/s41592-025-02833-w (PMC12510878; doi:10.1038/s41592-025-02833-w)
Supplement: Supplementary file 3 — Source code to run CaCoFold-R3D. Data and results. [file 41592_2025_2833_MOESM3_ESM.gz › supplemental_material/results/CaCoFold-R3D/CaCoFold-R3D_Rfam_vshuffle/RF02110_DLEU2_6.R2R.sto.pdf]

RF02110_DLEU2_6

5′- A A G C U C C R Y ● G A C U G C Y Y Y ● ● ● ● Y ● ● R ● R ● ● A U U U U Y R R ● R A ● ● U U U U ● ○ G Y G A U U U U U Y ● G Y G R R R U C Y U A R Y G G Y R C U R Y A C U C U C R G R C A G G U U A U C Y Y R U C U C U C C C G C U R G A U U G A U G A G A U C A G G C A U C
